# Prevalence and Clinical Relevance of Alström Syndrome Protein 1 Gene Variant and Feline Hypertrophic Cardiomyopathy in Sphynx Cats in Thailand

**DOI:** 10.3390/ani16121815

**Published:** 2026-06-12

**Authors:** Metita Sussadee, Thitichai Jarudecha, Rattana Muikaew, Korrawit Supaphom, Rucksak Rucksaken, Pratch Sukumolanan

**Affiliations:** Department of Veterinary Nursing, Faculty of Veterinary Technology, Kasetsart University, Bangkok 10900, Thailand; metita.sus@ku.th (M.S.); thitichai.j@ku.th (T.J.); rattana.m@ku.th (R.M.); korrawit212@gmail.com (K.S.); rucksak.r@ku.ac.th (R.R.)

**Keywords:** *ALMS1*, hypertrophic cardiomyopathy, Sphynx cats, echocardiography

## Abstract

Feline hypertrophic cardiomyopathy (HCM) is the most common cardiac disease in cats, causing morbidity and mortality. Recently, a variant in the Alström syndrome protein 1 (*ALMS1*) gene has been reported to be associated with HCM in Sphynx cats. However, information is limited on the prevalence and clinical significance of the *ALMS1* p.G2462R variant in Sphynx cats in Thailand. Therefore, the objectives of this study were to determine the prevalence of the *ALMS1* p.G2462R variant in Sphynx cats in Thailand and to assess its association with the clinical and echocardiographic features of feline HCM. A sample of 47 Sphynx cats was used based on specific inclusion and exclusion criteria. Clinical data, including sex, age, and body weight, together with echocardiographic assessments of the HCM phenotype and blood samples for *ALMS1* genotyping, were collected from each enrolled cat. The prevalence of the *ALMS1* p.G2462R variant was 44.68%, comprising 6.38% homozygous and 38.30% heterozygous mutations. Genotype frequencies were consistent with the Hardy–Weinberg equilibrium. However, no significant association was identified between the *ALMS1* p.G2462R variant and echocardiographic parameters related to the HCM phenotype. In conclusion, within this population, the *ALMS1* p.G2462R variant did not appear to play a primary role in the pathogenesis of HCM in Sphynx cats.

## Simple Summary

Hypertrophic cardiomyopathy (HCM) is a common and serious heart condition in cats. Recently, a specific genetic change in the Alström syndrome protein 1 (*ALMS*1) gene was suggested to be linked to this disease in Sphynx cats. However, information is limited on the prevalence and clinical significance of *ALMS1* mutations in Sphynx cats in Thailand. This was investigated using 47 Sphynx cats in this study according to the inclusion and exclusion criteria. Clinical data were obtained from all enrolled cats, including sex, age, and body weight, together with echocardiographic assessments of the HCM phenotype and blood samples for *ALMS1* genotyping. The prevalence of the *ALMS1* variant in this population was relatively high at 44.68%. However, no significant association was observed between the *ALMS1* variant and the HCM phenotype. These findings suggest that the *ALMS1* variant is unlikely to be the main cause of HCM in this Sphynx cat population.

## 1. Introduction

Hypertrophic cardiomyopathy (HCM) represents the most common cardiac disease in cats worldwide, with an estimated prevalence of 15% among the asymptomatic feline population [1,2]. This condition is characterized by concentric hypertrophy of the left ventricular myocardium without left ventricular chamber dilation, which impairs diastolic function and increases the risk of congestive heart failure, thromboembolism, and sudden cardiac death [3,4]. Diagnosing feline HCM is challenging because the disease often remains asymptomatic in its early stages [5,6]. Early detection depends primarily on echocardiography to assess cardiac morphology and function. Studies have demonstrated that echocardiographic identification of diastolic dysfunction is a valuable early diagnostic method for feline HCM [7,8].

The etiology of HCM is not fully understood. In humans, it is predominantly inherited in an autosomal dominant pattern [9,10]. Several pathogenic sarcomeric variants have been identified in association with familial human HCM. Numerous publications have indicated that the myosin-binding protein C gene (*MYBPC3*) and the β-myosin heavy chain gene (*MYH7*) are most frequently implicated [11,12,13,14].

Other non-sarcomeric genes have been proposed to contribute to cardiomyopathies in human medicine. Mutations in the Alström syndrome protein 1 (*ALMS1*) gene cause Alström syndrome, a rare autosomal recessive disorder. The *ALMS1* gene encodes a large protein of 4169 amino acids that is essential for the function of centrosome-associated sensory organelles, specifically primary cilia [15]. Clinically, Alström syndrome is characterized by metabolic disturbances, retinal dystrophy, sensorineural hearing loss, dilated cardiomyopathy (DCM), restrictive cardiomyopathy (RCM), and progressive fibrosis involving multiple organ systems [15,16]. Although *ALMS1*-related disease in humans is more commonly associated with DCM and RCM, *ALMS1* remains of interest in feline cardiology because the *ALMS1* mutation has previously been reported in association with HCM in Sphynx cats and proposed as a candidate variant in feline cardiomyopathy.

Over the past several decades, genetic studies of feline HCM have elucidated key aspects of its etiology, pathophysiology, and early detection for breeding purposes [10,17]. Furthermore, a mutation in the *ALMS1* gene has been identified as associated with left ventricular (LV) enlargement. Originally, the *ALMS1* p.G2462R (p.Gly2462Arg; c.7384G>C) variant was reported as a guanine-to-cytosine (G>C) substitution at position A3:92439157 in exon 12. Based on in silico analysis, this deleterious change was predicted to alter the protein’s structure from a coil to a helix, potentially affecting protein function. Furthermore, affected Sphynx cats with HCM and *ALMS1* gene mutations exhibit myofiber disarray, interstitial fibrosis, and increased nuclear proliferative activity [18]. However, data remain limited on the association between the *ALMS1* p.G2462R variant and the HCM phenotype across diverse Sphynx cat populations. Therefore, this study aimed to determine the prevalence of the *ALMS1* mutation in Sphynx cats in Thailand and to investigate its association with clinical and echocardiographic characteristics related to feline HCM.

## 2. Materials and Methods

### 2.1. Study Designs and Animals

This prospective cross-sectional study was conducted on Sphynx cats between April 2025 and June 2025. In total, 47 client-owned Sphynx cats were enrolled in this study based on predefined inclusion and exclusion criteria, with all 47 Sphynx cats being eligible for inclusion. However, some other prospective cats were excluded if they presented secondary left ventricular hypertrophy due to systemic hypertension (systolic blood pressure (SBP) exceeding 180 mmHg) or other cardiac abnormalities, such as aortic stenosis or other congenital or structural heart diseases. Additionally, cats that were pregnant, lactating, or had current or previous congestive heart failure (CHF) were excluded. Prior to study initiation, owner consent was obtained for all cats enrolled in this research.

### 2.2. Clinical Evaluation and Sample Collection

A clinical examination of each cat was performed by a licensed veterinarian. The signalment, including sex and age, at the time of evaluation, was recorded for each enrolled cat. Clinical data collected included body weight, body condition score (BCS), hydration status, heart rate (HR), respiratory rate (RR), heart sounds, and the presence of cardiac arrhythmia. Additionally, indirect blood pressure was measured using an oscillometric method, with SBP values recorded as the mean of three consecutive measurements.

A blood sample (approximately 2 mL per cat) was collected from the jugular, cephalic, or lateral saphenous veins. Minimal restraint was applied to reduce stress. The samples were transferred into ethylenediaminetetraacetic acid (EDTA) tubes and stored at −20 °C until laboratory analysis.

### 2.3. ALMS1 p.G2462R Genotyping

Genotyping of the *ALMS1* p.G2462R variant was performed based on extracting genomic DNA from 200 μL aliquots of each collected blood sample using an E.Z.N.A. Blood DNA Mini kit (Omega Bio-tek; Norcross, GA, USA) in accordance with the manufacturer’s instructions. DNA concentrations were quantified using a NanoDrop Lite Plus Spectrophotometer (Thermo Scientific; Waltham, MA, USA) prior to amplification. The oligonucleotide forward primer sequence was 5′-TCCCCTTCTGATCACACTG C-3′ and the reverse primer sequence was 5′-CCACTAGTCACCGCATGTCA-3′ [19,20]. Amplification was conducted using a T100 Thermal Cycler (Bio-Rad Laboratories; Hercules, CA, USA). The polymerase chain reaction (PCR) protocol consisted of an initial denaturation at 95 °C for 3 min, followed by 30 cycles of denaturation (95 °C for 30 s), annealing (60 °C for 30 s), and extension (72 °C for 1 min), with a final extension at 72 °C for 10 min. The PCR products from 242 bp were visualized on a 1.5% agarose gel. Subsequently, the PCR products were analyzed using Barcode Taq sequencing, and the results were evaluated using the A plasmid Editor (ApE) software version 3.1.7 (University of Utah, Salt Lake City, UT, USA).

### 2.4. Echocardiographic Examination and HCM Phenotyping

Transthoracic echocardiography was conducted to phenotype feline HCM. A single cardiac sonographer (P.S.) accomplished all echocardiographic examinations using a 4–10 MHz phased array transducer (Vetus E7, Mindray, Shenzhen, China). Accompanying Lead II electrocardiography was recorded during each echocardiographic assessment. For each subject, echocardiographic data were collected over three consecutive cardiac cycles. All examinations were performed without the use of sedation. In addition, all echocardiographic examinations were performed by a single operator using a standardized protocol. The operator was blinded to the genotyping results at the time of echocardiographic assessment.

The left ventricular condition was assessed using two-dimensional-guided M-mode echocardiography in the right parasternal short-axis view at the level of the chordae tendineae. In this plane, measurements consisted of intraventricular septal thickness (IVSd) or the end-diastolic left ventricular posterior wall thickness (LVPWd), end-diastolic left ventricular internal diameter (LVIDd), end-systolic intraventricular septal thickness (IVSs), end-systolic left ventricular posterior wall thickness (LVPWs), end-systolic left ventricular internal diameter (LVIDs), and percentage of LV fractional shortening (LV-FS%).

The assessment of the left atrium (LA) considered the left atrial-to-aortic root ratio (LA/AO), maximum left atrial diameter (LAD-max), and LA fractional shortening (LA-FS%). For the LA/AO ratio, LA and aortic diameters were measured in the right parasternal short-axis view of the heart base at the level of the aorta and left atrium immediately after aortic valve closure and before LA contraction. In the right parasternal four-chamber view, LAD-max was measured at end-systole, prior to mitral valve opening [21]. The contraction of LA was assessed based on the LA-FS%. The M-mode echocardiography was performed in the right parasternal long-axis LV outflow tract view. LA contraction was evaluated based on LA-FS%. M-mode echocardiography in the right parasternal transverse heart base at the aorta and left atrium view was measured based on the LA diameter at end-diastole (M-LAD) and end-systole (M-LAS). The percentage of LA fractional shortening was calculated as (LAD − LAS)/LAD) × 100 [22].

The transmitral inflow velocities were evaluated. The peak velocity of early-diastolic transmitral flow (E vel), the peak velocity of late-diastolic transmitral flow (A vel), and the E vel to A vel ratio (E/A) were measured from the left parasternal apical four-chamber view. Isovolumic relaxation time (IVRT) was measured in the left parasternal apical five-chamber view using pulse-wave Doppler echocardiography. Additionally, tissue Doppler imaging was performed to evaluate the myocardial velocities. Peak velocity of early diastolic mitral annular motion (E′ vel), peak velocity of late diastolic mitral annular motion (A′ vel), and the ratio of E vel to E′ vel (E/E′) were calculated.

According to the American College of Veterinary International Medicine (ACVIM) consensus statement guidelines for the classification, diagnosis, and management of cardiomyopathies in cats, feline HCM was diagnosed when either the IVSd or LVPWd exceeded 6.0 mm or when both measurements were greater than 6.0 mm without a dilated LV chamber [3]. The end-diastolic LV wall thickness between 5.5 mm and less than 6.0 mm is considered equivocal for HCM. Abnormalities associated with hypertrophic cardiomyopathy (HCM) were documented, including spontaneous echo contrast (SEC), systolic anterior motion (SAM) of the mitral valve leaflet, and left ventricular outflow tract obstruction (LVOTO). Subclinical HCM was staged as stated by ACVIM guidelines. All enrolled cats were classified as either stage A, indicating a predisposition to cardiomyopathy without evidence of myocardial disease, or stage B, indicating cardiomyopathy in the absence of clinical signs of CHF. Stage B cats were further subclassified into stage B1 or B2 based on left atrial size, with B1 representing cats at low risk and B2 representing those at higher risk of CHF and arterial thromboembolism.

### 2.5. Statistical Analysis

The data distribution was assessed using the Shapiro–Wilk test and histogram inspection. Normally distributed data are presented as the mean (standard deviation; SD), whereas non-normally distributed data are presented as the median (interquartile range; IQR). Categorical variables, including the number of cats, are presented as counts and percentages [*n* (%)]. Comparisons among the three *ALMS1* genotype groups were performed using one-way analysis of variance (ANOVA). Tukey’s post hoc test was used for normally distributed variables. The Kruskal–Wallis test followed by Dunn’s post hoc test was used for non-normally distributed variables. Categorical variables were compared using Fisher’s exact test. The Hardy–Weinberg equilibrium was used to evaluate genotype distribution. Homoscedasticity of residuals in linear regression models was evaluated. When heteroscedasticity was detected, regression analyses were performed using robust variance–covariance estimators to obtain valid standard errors. Multivariable linear regression was used to examine associations among clinical characteristics, echocardiographic parameters, and feline hypertrophic cardiomyopathy genotype, with regression coefficients (β) reported as mean differences and 95% confidence intervals (95% CI). Multivariable logistic regression was used to assess associations between characteristic variables and the presence of a positive feline HCM phenotype, with results expressed as odds ratios (ORs) and 95% CI. Additionally, the odds ratio for the association between *ALMS1* mutation status and the HCM phenotype was calculated. Statistical significance was set at *p* < 0.05. All analyses were conducted using STATA version 17.0 (StataCorp; College Station, TX, USA).

## 3. Results

### 3.1. Study Population and Genotype Distribution

In total, 47 Sphynx cats met the eligibility criteria and were enrolled between April and June 2025. Of these, 22 (46.81%) were male, and 25 (53.19%) were female. The median age was 24 months (IQR: 13–30). The mean body weight was 3.82 kg (SD: 0.94), and the median BCS was 5.75 (IQR: 5–6). Table 1 summarizes the clinical parameters of the enrolled Sphynx cats. The distributions of baseline demographic and signalment data, clinical characteristics, and echocardiographic characteristics of the enrolled Sphynx cats are provided in Supplementary Tables S1–S3.

Genotype distribution of the *ALMS1* p.G2462R variant was analyzed using PCR and Barcode Taq sequencing. Based on these results, the frequencies were 44.68% (21/47), comprising 6.38% (3/47) for the homozygous mutation (HOM), 38.30% (18/47) for the heterozygous mutation (HET), and 55.32% (26/47) for the homozygous wild-type (WT). The Hardy–Weinberg equilibrium was evaluated to determine whether the observed genotype frequencies compared to the expected frequencies within the study population. The genotype distribution was consistent with the Hardy–Weinberg equilibrium (χ^2^ = 0.043, *p* = 0.836), indicating that the study population was in genetic equilibrium (Table 2).

The comparison of clinical parameters among the three genotype groups of the *ALMS1* p.G2462R mutation is presented in Table 1. Based on these results, there were no significant differences in sex, age, body weight, BCS, HR, RR, or SBP among these groups.

### 3.2. Echocardiographic Findings and HCM Phenotype

**Figure 1:**
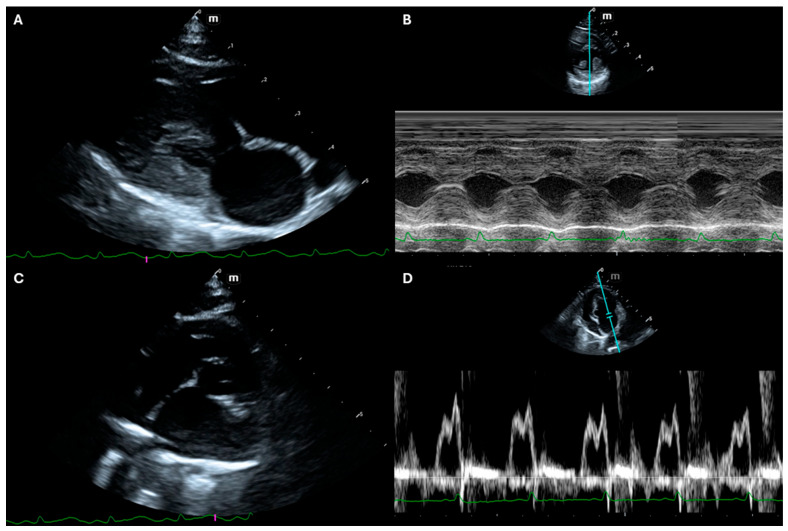
Representative echocardiographic findings in a Sphynx cat diagnosed with HCM. Right parasternal long-axis four-chamber view demonstrating LA enlargement and LV wall thickening (**A**). M-mode echocardiography obtained from the right parasternal short-axis view at the chordae tendineae level, illustrating LV hypertrophy consistent with the HCM phenotype (**B**). B-mode echocardiography obtained from the right parasternal short-axis view at the level of the aorta and left atrium during early diastole, showing measurement of LA enlargement using the LA/AO ratio (**C**). Transmitral inflow velocity pattern obtained from the left parasternal apical four-chamber view for evaluation of diastolic function (**D**).

Echocardiography assessment revealed cardiac structural and functional characteristics. The overall prevalence of the HCM phenotype among the enrolled cats was 8.51% (4/47). Specifically, the HCM phenotype was identified in 11.11% (2/18) of heterozygous (HET) cats and 7.69% (2/26) of homozygous wild-type (WT) cats. However, there were no significant differences in the echocardiographic parameters among the different *ALMS1* gene mutation groups. According to the ACVIM staging system for feline cardiomyopathies, two *ALMS1* mutation cats were classified as stage B1, while two *ALMS1* wild-type cats were classified as stage B2. Echocardiographic data for the enrolled cats are provided in Table 3, and a representative echocardiographic image of a Sphynx cat with HCM is displayed in Figure 1.

### 3.3. Clinical and Genotypic Associations with Echocardiographic Parameters and HCM Phenotype

Multivariable linear regression analysis with robust variance–covariance estimators was used to evaluate the associations between clinical variables and echocardiographic parameters. Mature Sphynx cats were significantly associated with increased values for IVSd (β = 0.14, 95% CI: 0.10–0.18, *p* < 0.001), LVPWd (β = 0.16, 95% CI: 0.12–0.21, *p* < 0.001), the LA/AO ratio (β = 0.24, 95% CI: 0.12–0.36, *p* < 0.001), and IVRT (β = 6.57, 95% CI: 2.49–10.64, *p* = 0.002), while the MV E/A (β = −0.50, 95% CI: −0.71 to −0.29, *p* < 0.001) and MV E/E′ (β = −4.26, 95% CI: −5.75 to −2.77, *p* < 0.001) ratios were significantly decreased compared with the junior group after adjustment for sex and BCS. In contrast, kittens were associated with lower values for IVSd (β = −0.05, 95% CI: −0.09 to 0.00, *p* = 0.035) and the LA/AO ratio (β = −0.21, 95% CI: −0.34 to −0.09, *p* = 0.001), while the MV E/E′ ratio significantly increased (β = 11.91, 95% CI: 10.07–13.75, *p* < 0.001) compared to the junior group after adjustment for sex and BCS. Compared to cats with an ideal BCS, the underweight Sphynx cats had a significantly higher MV E/E′ ratio (β = 6.48, 95% CI: 2.80–10.16, *p* = 0.001) after adjustment for sex and age (Table 4). Sex was not a significant predictor in the model. Multivariable analyses of echocardiographic parameters in relation to clinical presentation are presented in Table 4.

Multivariable linear regression analyses were conducted to assess the associations between echocardiographic parameters (IVSd, LVPWd, IVRT, LA/AO ratio, MV E/A ratio, and MV E/E′ ratio) and clinical characteristics (systolic blood pressure, heart rate, and respiratory rate), with adjustments for age and body weight (Table 5). According to these analyses, each 1-unit increase in systolic blood pressure was associated with a 0.82-unit decrease in IVRT (95% CI: −1.58 to −0.06, *p* = 0.034). Furthermore, compared to the WT group, Sphynx cats in the HOM group had significantly decreased IVSd (β = −0.08, 95% CI: −0.12–−0.04, *p* <0.001) and IVRT (β = −7.84, 95% CI: −11.38–−4.30, *p* <0.001) after adjustment for age, as displayed in Table 6.

The association between the HCM phenotype and *ALMS1* mutation status was evaluated using the odds ratio (OR). The estimated OR was 1.26 (95% CI: 0.16–9.82); however, this association was not significant (*p* = 0.823). Additionally, in the multivariable logistic regression analysis, sex (OR = 1.62, 95% CI: 0.11–22.77, *p* = 0.720), age (OR = 3.78, 95% CI: 0.29–49.37, *p* = 0.310), and BCS (OR = 2.03, 95% CI: 0.14–29.44, *p* = 0.604) were not significantly associated with a positive HCM phenotype (Supplementary Table S4). Therefore, based on these results, there was no significant association between *ALMS1* mutation status or the evaluated clinical variables and the presence of the HCM phenotype in this Sphynx cat population.

## 4. Discussion

This study provided the first report of *ALMS1* p.G2462R mutation prevalence in Sphynx cats in Thailand and assessed its clinical association with the HCM phenotype. Additionally, a meticulous assessment of the associations between echocardiographic parameters and clinical variables, including sex, age, and BCS, was performed.

Based on the results, the prevalence of the *ALMS1* p.G2462R mutation in Sphynx cats in Thailand was 55.32% for the homozygous wild-type, 38.30% for the heterozygous mutation, and 6.38% for the homozygous mutation. This finding was consistent with other reports indicating a relatively high prevalence of the *ALMS1* mutation in Sphynx cats, with estimated frequencies of approximately 50–70% reported across multiple geographic regions, including New Zealand, Japan, the USA, and Europe [18,19,20,23,24].

The initial report describing the association between *ALMS1* mutations and HCM suggested a breed-specific occurrence in Sphynx cats [18]. However, subsequent studies have demonstrated that *ALMS1* mutations are not limited to this breed. For example, a study from Japan reported *ALMS1* mutations in Munchkin and Scottish Fold cats [24], while investigations in the USA and Europe have identified these mutations in multiple breeds, including the British Shorthair, British Longhair, Ragdoll, Sphynx, Maine Coon, and Devon Rex [19]. This observation may be explained by their genetic relationship. Notably, the Sphynx and Devon Rex breeds have a documented history of crossbreeding, and the *ALMS1* mutation has been identified in Devon Rex cats with an allele frequency of 32.81% [19].

In humans, mutations in the *ALMS1* gene result in Alström syndrome, a rare inherited multisystem disorder that affects several organs, including the retina, inner ear, liver, kidneys, endocrine system, and heart [25,26]. While there has been no clear identification of a correlation between genotype and phenotype regarding *ALMS1* gene mutations and specific clinical characteristics, various studies have demonstrated that *ALMS1* gene mutations are associated with the development of severe cardiomyopathies, DCM, and RCM [27,28,29]. Among human patients with Alström syndrome who develop cardiomyopathies, approximately one-third present during infancy. Notably, human infantile DCM may resolve completely, with normal cardiac function reversible within 2–3 years [29,30]. Furthermore, a recent study involving a large Chinese population demonstrated that a truncating *ALMS1* variant in exon 16 was significantly increased in *ALMS*-associated cardiomyopathy in infants [29]. Echocardiographic assessments have identified both LV systolic and diastolic impairment in affected children with cardiomyopathy. However, a study investigating DCM caused by *ALMS1* mutations reported inconsistent disease severity among siblings carrying similar *ALMS1* mutations, indicating that modifier genes or environmental factors may contribute to phenotypic variability [29,30,31,32,33]. In contrast, RCM typically develops during adolescence and adulthood in patients with *ALMS1* mutations. Fibrosis and pulmonary hypertension are established features of RCM associated with *ALMS1* mutations. Nevertheless, current knowledge remains limited regarding RCM in individuals with *ALMS1* mutations [28,33]. Although *ALMS1*-related disease in humans is more commonly associated with DCM, no cats with a DCM phenotype were identified in the present cohort. Future studies examining the potential role of the *ALMS1* p.G2462R variant in feline DCM may provide additional clinically relevant insights. In addition, although *ALMS1*-related disease is well characterized in humans, a clearly defined Alström syndrome or Alström-like syndrome has not yet been established in cats. Therefore, in feline medicine, *ALMS1* should currently be regarded as a candidate gene of interest rather than a confirmed cause of cardiomyopathy.

In contrast, although another study has implicated *ALMS1* variants in Sphynx cats [18], the current investigation did not identify a significant association between *ALMS1* mutation status and the HCM phenotype. This absence of association was observed consistently across echocardiographic analyses, multivariable logistic regression, and odds ratio estimation. The echocardiographic assessment revealed that 8.51% of the enrolled Sphynx cats had HCM, which was lower than the approximately 15% prevalence reported in other studies among apparently healthy cats with increasing age [1,2]. This lower prevalence could be partially explained by the relatively young age of the enrolled cats (median age of 24 months (IQR: 13–30)). In the current study, no significant differences in echocardiographic characteristics were identified among the three *ALMS1* mutation groups. According to the multivariable linear regression analyses, sex was not associated with any echocardiographic alterations. This result contrasted with another report based on Sphynx cats in New Zealand, which found that the male sex was significantly associated with HCM in baseline group comparisons [23].

In the general feline population, HCM is an age-dependent disease [1,34,35]. In line with this, age was one of the major determinants of echocardiographic variation in the current study, being associated with increased left ventricular wall thickness, left atrial enlargement, and prolonged IVRT, as well as lower MV E/A and MV E/E′ ratios. This result differed from another report in New Zealand, where there was no significant association between age and echocardiographic parameters in the longitudinal study. Therefore, age should be considered carefully when interpreting echocardiographic findings and assessing the risk of HCM in cats. Additionally, the current analysis identified an association between elevated systolic blood pressure and increased IVRT. In humans, this association may result in impaired left ventricular relaxation or LV hypertrophy and remodeling [36]. However, further studies in cats are necessary to confirm these findings.

The genotype analysis demonstrated that homozygous mutant cats had significantly lower IVSd and IVRT compared to WT cats, which contrasted with the alterations typically observed in HCM [3,37]. No significant genotype differences were identified for LVPWd, the LA/AO ratio, the MV E/A ratio, or the MV E/E′ ratio. Altogether, these findings indicated that the *ALMS1* p.G2462R variant was not associated with an HCM phenotype in this cohort.

Although the result was close to 1 for the estimated OR for the *ALMS1* mutation and HCM development, and the multivariable logistic regression for the association of clinical variables with the HCM phenotype, no significant association was identified. This finding suggested that *ALMS1* mutations were unlikely to be associated with HCM development in this population, potentially due to the limited number of HCM-positive cats. The current findings were consistent with other studies in New Zealand, the USA, and Europe [19,23]. In the present study, none of the three cats carrying the homozygous mutant *ALMS1* p.G2462R genotype exhibited an HCM phenotype, whereas HCM was identified only in heterozygous and wild-type cats. Therefore, our findings do not support a clear genotype–phenotype association between the homozygous *ALMS1* p.G2462R variant and HCM in this cohort. This observation may reflect low or variable disease penetrance, whereby the presence of the variant does not consistently lead to phenotypic expression. By contrast, this finding should be interpreted with caution because of the limited number of homozygous cats and HCM-positive cases. However, these results differed from those in the initial publication, which reported a relative risk of 13.6 in the feline population [18]. These discrepancies may be attributed to differences in geographical region, study period, and feline population characteristics.

In the current study, the analysis of the Hardy–Weinberg equilibrium demonstrated that the observed genotype frequencies were consistent with expected frequencies, indicating genetic stability in the study population. This contrasts with other research on Maine Coon cats with the *MYBPC*3 p.A31P mutation, which identified deviations from the Hardy–Weinberg equilibrium and suggested reduced survival in homozygotes [38,39]. Consequently, these findings indicated that the *ALMS1* p.G2462R variant was unlikely to be under stable genetic selection and may not significantly affect survival due to population bias or genotyping error.

The absence of a significant association between the *ALMS1* p.G2462R variant and the HCM phenotype in the current study’s cohort of Sphynx cats may be explained by several mechanisms, including incomplete penetrance. Although the *ALMS1* p.G2462R variant has previously been implicated in cardiomyopathy, not all individuals carrying the mutation develop a detectable HCM phenotype—a phenomenon well documented in both human and feline cardiomyopathies, where genetic variants may predispose to disease without consistent phenotypic expression [9,10].

Genetic heterogeneity is also likely to be a significant contributing factor. Feline HCM is recognized as a complex, genetically diverse disease involving multiple genes and variants in its pathogenesis. In the context of genetic panels commonly used for testing, while sarcomeric genes, such as *MYBPC3* p.A31P in Maine Coon cats and *MYBPC3* p.R820W in Ragdoll cats, are well-established contributors, non-sarcomeric genes such as *ALMS1* may exert more modest or context-dependent effects [11,12,13,14,17].

In addition, the phenotypic expression of cardiomyopathy-related genes can be significantly influenced by interactions among multiple loci. Phenotypic variability has been reported among individuals carrying similar *ALMS1* mutations, suggesting a role for modifier genes and epistatic interactions [30]. Furthermore, environmental and physiological factors, such as blood pressure and metabolic status, may modulate cardiac structure and function [35,36]. In the current study, the observed associations between age, body condition score, and echocardiographic parameters support the conclusion that non-genetic factors contribute substantially to cardiac variation, potentially overshadowing the effect of a single genetic variant.

Several limitations in the current study should be acknowledged. First, HCM should be regarded as a diagnosis of exclusion. Although cats with obvious secondary causes of myocardial hypertrophy were clinically excluded, undetected systemic conditions cannot be completely ruled out. Thyroid status and renal function were not routinely assessed in this cohort because the enrolled cats were relatively young. Second, the relatively small sample size, combined with the limited number of homozygous mutant cats and HCM-positive cases, may have reduced the statistical power of this study to detect a modest association between the *ALMS1* p.G2462R variant and the HCM phenotype. Therefore, the absence of a statistically significant association should be interpreted with caution. In addition, all echocardiographic examinations were performed by a single operator. Although this approach reduced inter-observer variability and the operator was blinded to the genotyping results, operator-related measurement bias cannot be completely excluded. Third, the cross-sectional design and young age distribution prevented the assessment of disease progression, temporal relationships, and late-onset penetrance. Fourth, restriction to a single breed of Sphynx cat may limit the generalizability of these findings. Fifth, the analysis focused solely on a single *ALMS1* p.G2462R variant. Genetic heterogeneity in feline HCM suggests that unmeasured variants and polygenic contributions may play a substantial role in Sphynx cats. Future studies are necessary, with larger, multi-breed cohorts and longitudinal follow-up to further elucidate the genetic characterization of feline HCM.

## 5. Conclusions

This study provided important insights into the prevalence of the *ALMS1* p.G2462R variant and the factors influencing echocardiographic characteristics and HCM in Sphynx cats in Thailand. No significant association was identified between the *ALMS1* p.G2462R variant and the HCM phenotype, although several factors influencing echocardiographic variation were observed. These findings emphasize that the *ALMS1* p.G2462R variant is unlikely to be a major determinant of HCM in this population. Nevertheless, the present findings provide a useful basis for future studies examining the genetic background of feline HCM. At present, analysis of the *ALMS1* p.G2462R variant cannot replace echocardiographic examination, particularly because myocardial hypertrophy in cats is heterogeneous and is likely influenced by multiple genetic and non-genetic factors.

## Figures and Tables

**Table 1:** Comparison of clinical parameters among three genotype groups of *ALMS1* p.G2462R mutation. Abbreviation: HET, heterozygous mutation; HOM, homozygous mutation; SBP, systolic blood pressure; WT, homozygous wild-type.

Clinical Variable	Total (*n* = 47)	*ALMS1* p.G2462R WT Group (*n* = 26)	*ALMS1* p.G2462R HET Group (*n* = 18)	*ALMS1* p.G2462R HOM Group (*n* = 3)	*p* Value
Sex					0.3708
–Female, *n* (%)	25 (53.19%)	16 (61.54%)	7 (38.89%)	2 (66.67%)	
–Male, *n* (%)	22 (46.81%)	10 (38.46%)	11 (61.11%)	1 (33.33%)	
Age (month), median (IQR)	24 (13–30)	21 (19.25–30)	20.5 (11–31.5)	10 (9–14)	0.1116
Body weight (kg), mean (SD)	3.82 (0.94)	3.83 (0.19)	3.91 (0.23)	3.18 (0.27)	0.4694
BCS, median (IQR)	5.75 (5–6)	5 (5–6)	6 (5–7)	5 (5–5.5)	0.1758
HR (beat/minute), mean (SD)	202.23 (6.90)	205.27 (8.95)	205.61 (10.90)	157.67 (34.74)	0.2292
RR (breath/minute), mean (SD)	52.05 (2.27)	50.30 (2.88)	56.41 (3.92)	40.67 (4.67)	0.3647
SBP (mmHg), mean (SD)	152.18 (3.99)	148.10 (4.51)	160.90 (6.50)	138.08 (31.52)	0.2082

**Table 2:** Observed and expected allele frequencies in the Sphynx breed under the Hardy–Weinberg equilibrium. Abbreviation: HET, heterozygous mutation; HOM, homozygous mutation; WT, homozygous wild-type.

Genotyping	Observed	Expected	*p* Value
WT	26	26.06	0.9609
HET	18	17.87	
HOM	3	3.06	
Total	47	47	

**Table 3:** Echocardiographic characteristics of Sphynx cats in three *ALMS1* genotypic groups. Abbreviations: IVRT, isovolumic relaxation time; IVSd, interventricular septum thickness at end-diastole; IVSs, interventricular septum thickness at end-systole; LA/AO ratio, left atrial and aorta ratio; LAD max, maximum left atrial diameter; LA-FS, left atrial fractional shortening; LV-FS, left ventricular fractional shortening; LVIDd, left ventricular internal dimension at end-diastole; LVIDs, left ventricular internal dimension at end-systole; LVPWd, left ventricular posterior wall thickness at end-diastole; LVPWs, left ventricular posterior wall thickness at end-systole; MV A vel, peak velocity of early diastolic transmitral flow; MV E vel, peak velocity of early diastolic transmitral flow; MV E/A ratio, ratio of E to A; MV E/E′ ratio, ratio of E to E′; MV E′ vel, peak velocity of early diastolic mitral annular motion.

Echocardiographic Variable	*ALMS1* p.G2462R WT Group (*n* = 26)	*ALMS1* p.G2462R HET Group (*n* = 18)	*ALMS1* p.G2462R HOM Group (*n* = 3)	*p* Value
IVSd (cm), mean (SD)	0.46 (0.01)	0.47 (0.02)	0.35 (0.01)	0.0546
LVIDd (cm), mean (SD)	1.69 (0.05)	1.58 (0.05)	1.78 (0.14)	0.2746
LVPWd (cm), mean (SD)	0.47 (0.02)	0.45 (0.02)	0.38 (0.03)	0.2027
IVSs (cm), mean (SD)	0.61 (0.02)	0.65 (0.02)	0.56 (0.06)	0.2351
LVIDs (cm), mean (SD)	1.02 (0.06)	0.86 (0.04)	1.04 (0.06)	0.1162
LVPWs (cm), mean (SD)	0.56 (0.02)	0.61 (0.04)	0.48 (0.04)	0.2128
LV-FS (%), mean (SD)	44.43 (1.74)	45.47 (1.94)	41.28 (1.66)	0.1481
LAD max (cm), mean (SD)	1.30 (0.03)	1.26 (0.04)	1.26 (0.06)	0.7633
LA/AO (2D), mean (SD)	1.46 (0.04)	1.38 (0.04)	1.49 (0.10)	0.4203
LA-FS (%), mean (SD)	44.43 (1.39)	47.50 (2.37)	41.28 (1.66)	0.2430
MV E vel (cm/s), mean (SD)	89.72 (2.97)	87.37 (5.34)	93.01 (10.52)	0.8532
MV A vel (cm/s), mean (SD)	76.48 (4.47)	77.58 (5.64)	82.43 (17.05)	0.9158
MV E/A ratio, mean (SD)	1.16 (0.05)	1.22 (0.13)	0.87 (0.16)	0.3747
MV E′ vel (cm/s), mean (SD)	10.43 (0.67)	11.08 (0.94)	10.88 (1.26)	0.9754
MV E/E′ ratio, mean (SD)	9.45 (0.62)	11.11 (0.94)	8.88 (2.19)	0.8996
IVRT (ms), mean (SD)	47.13 (2.57)	42.37 (1.31)	36.6 (0.6)	0.1410
HCM, *n* (%)	2 (7.69%)	2 (11.11%)	-	-

**Table 4:** Multivariable linear regression analysis with robust variance–covariance estimators assessing associations between clinical variables and echocardiographic parameters. Abbreviations: IVSd, interventricular septum thickness at end-diastole; LA/AO ratio, left atrial and aorta ratio; LVPWd, left ventricular posterior wall thickness at end-diastole; IVRT, isovolumic relaxation time; MV E/A ratio, ratio of E to A; MV E/E′ ratio, ratio of E to E′.

Clinical Variable	Category	IVSd β (95% CI)	*p* Value	LVPWd β (95% CI)	*p* Value	LA/AO β (95% CI)	*p* Value	IVRT β (95% CI)	*p* Value	MV E/A β (95% CI)	*p* Value	MV E/E′ β (95% CI)	*p* Value
Sex	Female	Reference	–	Reference	–	Reference	–	Reference	–	Reference	–	Reference	–
	Male	0.02 (−0.02–0.07)	0.295	0.02 (−0.03–0.08)	0.349	−0.08 (−0.21–0.05)	0.229	3.14 (−5.82–12.11)	0.482	0.20 (0–0.41)	0.053	−0.36 (−1.85–1.13)	0.629
Age group	Junior	Reference	–	Reference	–	Reference	–	Reference	–	Reference	–	Reference	–
	Adult	0.04 (−0.01–0.09)	0.104	0.07 (0.00–0.14)	0.040	−0.03 (−0.17–0.11)	0.653	2.60 (−4.32–9.53)	0.452	0.12 (−0.17–0.42)	0.398	−0.32 (−2.39–1.74)	0.754
	Mature	0.14 (0.10–0.18)	<0.001	0.16 (0.12–0.21)	<0.001	0.24 (0.12–0.36)	<0.001	6.57 (2.49–10.64)	0.002	−0.50 (−0.71–−0.29)	<0.001	−4.26 (−5.75–−2.77)	<0.001
	Kitten	−0.05 (−0.09–0.00)	0.035	0 (−0.05–0.05)	0.963	−0.21 (−0.34–−0.09)	0.001	−3.00 (−7.71–1.71)	0.206	−0.07 (−0.26–0.12)	0.446	11.91 (10.07–13.75)	<0.001
BCS	Ideal	Reference	–	Reference	–	Reference	–	Reference	–	Reference	–	Reference	–
	Overweight	0 (−0.06–0.05)	0.880	0.03 (−0.03–0.91)	0.351	0.07 (−0.06–0.21)	0.280	−5.71 (−14.74–3.32)	0.208	0.10 (−0.14–0.35)	0.402	0.00 (−1.89–1.89)	1.000
	Underweight	−0.01 (−0.06–0.05)	0.735	−0.04 (−0.15–0.07)	0.495	0.08 (−0.07–0.23)	0.305	−2.30 (−8.21–3.61)	0.436	0.11 (−0.01–0.24)	0.081	6.48 (2.80–10.16)	0.001

**Table 5:** Multivariable linear regression analyses with robust variance–covariance estimators assessing associations between echocardiographic parameters and clinical characteristics of cats, adjusted for age and body weight. Abbreviations: IVRT, isovolumic relaxation time; IVSd, interventricular septum thickness at end-diastole; LA/AO ratio, left atrial and aorta ratio; LVPWd, left ventricular posterior wall thickness at end-diastole; MV E/A ratio, ratio of E to A; MV E/E′ ratio, ratio of E to E′.

Echocardiographic Variable	Systolic Blood Pressure β (95% CI)	*p* Value	Heart Rate * β (95% CI)	*p* Value	Respiratory Rate β (95% CI)	*p* Value
IVSd	−26.65 (−157.08–103.79)	0.682	12.66 (−216.84–242.16)	0.912	72.83 (0.31–145.34)	0.049
LVPWd	−9.63 (−103.20–83.93)	0.836	9.20 (−181.15–199.55)	0.923	25.22 (−27.29–77.73)	0.337
IVRT	−0.82 (−1.58–−0.06)	0.034	−0.46 (−1.20–0.28)	0.216	−0.16 (−0.62–0.30)	0.493
LA/AO ratio	−21.92 (−62.66–18.82)	0.284	−15.10 (−100.80–70.59)	0.724	−8.36 (−32.83–16.12)	0.494
MV E/A ratio	4.86 (−16.27–26.00)	0.645	−1.84 (−20.23–16.56)	0.841	1.07 (−11.14–13.27)	0.860
MV E/E′ ratio	−0.90 (−3.94–2.14)	0.553	12.66 (−7.59–0.60)	0.092	0.88 (−0.45–2.21)	0.188

* Multivariable linear regression with robust variance–covariance estimators.

**Table 6:** Multivariable linear regression analyses with robust variance–covariance estimators assessing the associations between echocardiographic parameters and *ALMS1* genotypes, using WT and HET as the reference group and adjusting for age (months). Abbreviation: HET, heterozygous mutation; HOM, homozygous mutation; IVRT, isovolumic relaxation time; IVSd, interventricular septum thickness at end-diastole; LA/AO ratio, left atrial and aorta ratio; LVPWd, left ventricular posterior wall thickness at end-diastole; MV E/A ratio, ratio of E to A; MV E/E′ ratio, ratio of E to E′; WT, homozygous wild-type.

Variable	Category	IVSd β (95% CI)	*p* Value	LVPWd β (95% CI)	*p* Value	LA/AO β (95% CI)	*p* Value	IVRT β (95% CI)	*p* Value	MV E/A β (95% CI)	*p* Value	MV E/E′ β (95% CI)	*p* Value
Genotype	WT	Reference	–	Reference	–	Reference	–	Reference	–	Reference	–	Reference	–
	HET	0.01 (−0.04–0.06)	0.736	−0.02 (−0.08–0.04)	0.465	−0.07 (−0.19–0.04)	0.201	−4.77 (−10.65–1.11)	0.109	0.07 (−0.21–0.35)	0.632	−0.73 (−3.24–1.77)	0.559
	HOM	−0.10 (−0.14–−0.07)	<0.001	−0.09 (−0.15–0.02)	0.012	0.03 (−0.16–0.22)	0.777	−10.53 (−15.88–−5.19)	<0.001	−0.28 (−0.57–0.01)	0.058	0.23 (−5.18–5.64)	0.932

## Data Availability

The data presented in this study are available from the corresponding author upon reasonable request. The data are not publicly available due to privacy and ethical restrictions related to client-owned animals.

## Supplementary Materials

The following supporting information can be downloaded at: https://www.mdpi.com/article/10.3390/ani16121815/s1. Table S1: baseline demographic and signalment data of the enrolled Sphynx cats; Table S2: distribution of clinical characteristics according to sex, age group, and body condition score; Table S3: distribution of echocardiographic characteristics and positive HCM phenotype according to sex, age group, and body condition score; Table S4: Analysis of multivariable logistic regression for relation of characteristic variables to positive phenotype.

## References

[1] Payne J.R., Brodbelt D.C., Luis Fuentes V. (2015). Cardiomyopathy prevalence in 780 apparently healthy cats in rehoming centres (the CatScan study). J. Vet. Cardiol..

[2] Paige C.F., Abbott J.A., Elvinger F., Pyle R.L. (2009). Prevalence of cardiomyopathy in apparently healthy cats. J. Am. Vet. Med. Assoc..

[3] Luis Fuentes V., Abbott J., Chetboul V., Côté E., Fox P.R., Häggström J., Kittleson M.D., Schober K., Stern J.A. (2020). ACVIM consensus statement guidelines for the classification, diagnosis, and management of cardiomyopathies in cats. J. Vet. Intern. Med..

[4] Häggström J., Luis Fuentes V., Wess G. (2015). Screening for hypertrophic cardiomyopathy in cats. J. Vet. Cardiol..

[5] Payne J., Luis Fuentes V., Boswood A., Connolly D., Koffas H., Brodbelt D. (2010). Population characteristics and survival in 127 referred cats with hypertrophic cardiomyopathy (1997 to 2005). J. Small Anim. Pract..

[6] Payne J., Borgeat K., Connolly D., Boswood A., Dennis S., Wagner T., Menaut P., Maerz I., Evans D., Simons V. (2013). Prognostic indicators in cats with hypertrophic cardiomyopathy. J. Vet. Intern. Med..

[7] Sugimoto K., Fujii Y., Sunahara H., Aoki T. (2015). Assessment of left ventricular longitudinal function in cats with subclinical hypertrophic cardiomyopathy using tissue Doppler imaging and speckle tracking echocardiography. J. Vet. Med. Sci..

[8] Koffas H., Dukes-McEwan J., Corcoran B., Moran C., French A., Sboros V., Simpson K., McDicken W. (2006). Pulsed tissue Doppler imaging in normal cats and cats with hypertrophic cardiomyopathy. J. Vet. Intern. Med..

[9] Kittleson M.D., Meurs K.M., Harris S.P. (2015). The genetic basis of hypertrophic cardiomyopathy in cats and humans. J. Vet. Cardiol..

[10] Walsh R., Offerhaus J.A., Tadros R., Bezzina C.R. (2022). Minor hypertrophic cardiomyopathy genes, major insights into the genetics of cardiomyopathies. Nat. Rev. Cardiol..

[11] Franke M., Książczyk T.M., Dux M., Chmielewski P., Truszkowska G., Czapczak D., Pietrzak R., Bilinska Z.T., Demkow U., Werner B. (2024). A *MYH7* variant in a five-generation-family with hypertrophic cardiomyopathy. Front. Genet..

[12] Mancuso G., Marsan M., Neroni P., Soddu C., Lai F., Serventi L., Cau M., Coiana A., Incani F., Murru S. (2025). Clinical and Genetic Heterogeneity of HCM: The Possible Role of a Deletion Involving *MYH6* and *MYH7*. Genes.

[13] Tudurachi B.-S., Zăvoi A., Leonte A., Țăpoi L., Ureche C., Bîrgoan S.G., Chiuariu T., Anghel L., Radu R., Sascău R.A. (2023). An update on *MYBPC3* gene mutation in hypertrophic cardiomyopathy. Int. J. Mol. Sci..

[14] Maron B.J., Maron M.S., Semsarian C. (2012). Genetics of hypertrophic cardiomyopathy after 20 years: Clinical perspectives. J. Am. Coll. Cardiol..

[15] Hearn T. (2019). *ALMS1* and Alström syndrome: A recessive form of metabolic, neurosensory and cardiac deficits. J. Mol. Med..

[16] Hearn T., Renforth G.L., Spalluto C., Hanley N.A., Piper K., Brickwood S., White C., Connolly V., Taylor J.F., Russell-Eggitt I. (2002). Mutation of *ALMS1*, a large gene with a tandem repeat encoding 47 amino acids, causes Alström syndrome. Nat. Genet..

[17] Grzeczka A., Graczyk S., Pasławski R., Pasławska U. (2024). Genetic basis of hypertrophic cardiomyopathy in cats. Curr. Issues Mol. Biol..

[18] Meurs K.M., Williams B.G., DeProspero D., Friedenberg S.G., Malarkey D.E., Ezzell J.A., Keene B.W., Adin D.B., DeFrancesco T.C., Tou S. (2021). A deleterious mutation in the *ALMS1* gene in a naturally occurring model of hypertrophic cardiomyopathy in the Sphynx cat. Orphanet J. Rare Dis..

[19] Boeykens F., Abitbol M., Anderson H., Dargar T., Ferrari P., Fox P.R., Hayward J.J., Häggström J., Davison S., Kittleson M.D. (2024). Classification of feline hypertrophic cardiomyopathy-associated gene variants according to the American College of Medical Genetics and Genomics guidelines. Front. Vet. Sci..

[20] Turba M.E., Ferrari P., Milanesi R., Gentilini F., Longeri M. (2023). HCM-associated *ALMS1* variant: Allele drop-out and frequency in Italian Sphynx cats. Anim. Genet..

[21] Duler L., Scollan K., LeBlanc N. (2019). Left atrial size and volume in cats with primary cardiomyopathy with and without congestive heart failure. J. Vet. Cardiol..

[22] Machado A., Partington C., Silva J., Gardner L., Matos J.N. (2024). Left atrial fractional shortening in cats: A comparison between two echocardiographic views. J. Vet. Cardiol..

[23] Seo J., Loh Y., Connolly D.J., Luis Fuentes V., Dutton E., Hunt H., Munday J.S. (2024). Prevalence of Hypertrophic Cardiomyopathy and *ALMS1* Variant in Sphynx Cats in New Zealand. Animals.

[24] Akiyama N., Suzuki R., Saito T., Yuchi Y., Ukawa H., Matsumoto Y. (2023). Presence of known feline *ALMS1* and *MYBPC3* variants in a diverse cohort of cats with hypertrophic cardiomyopathy in Japan. PLoS ONE.

[25] Brofferio A., Sachdev V., Hannoush H., Marshall J.D., Naggert J.K., Sidenko S., Noreuil A., Sirajuddin A., Bryant J., Han J.C. (2017). Characteristics of cardiomyopathy in Alström syndrome: Prospective single-center data on 38 patients. Mol. Genet. Metab..

[26] Bea-Mascato B., Valverde D. (2024). Genotype–phenotype associations in Alström syndrome: A systematic review and meta-analysis. J. Med. Genet..

[27] Dedeoglu S., Dede E., Oztunc F., Gedikbasi A., Yesil G., Dedeoglu R. (2022). Mutation identification and prediction for severe cardiomyopathy in Alström syndrome, and review of the literature for cardiomyopathy. Orphanet J. Rare Dis..

[28] Ozantürk A., Marshall J.D., Collin G.B., Düzenli S., Marshall R.P., Candan Ş., Tos T., Esen İ., Taşkesen M., Çayır A. (2015). The phenotypic and molecular genetic spectrum of Alström syndrome in 44 Turkish kindreds and a literature review of Alström syndrome in Turkey. J. Hum. Genet..

[29] Huang Y., Wang L., Zhang Q., Gao S., Chang G., Yu T., Yao R.-e., Ding Y., Wang X. (2025). Alström syndrome: A cross-sectional and follow-up study of 127 patients in China, highlighting genetic variant spectrum and cardiac features. Orphanet J. Rare Dis..

[30] Mahamid J., Lorber A., Horovitz Y., Shalev S.A., Collin G.B., Naggert J.K., Marshall J.D., Spiegel R. (2013). Extreme clinical variability of dilated cardiomyopathy in two siblings with Alström syndrome. Pediatr. Cardiol..

[31] Loudon M.A., Bellenger N.G., Carey C.M., Paisey R.B. (2009). Cardiac magnetic resonance imaging in Alström syndrome. Orphanet J. Rare Dis..

[32] Marshall J.D., Muller J., Collin G.B., Milan G., Kingsmore S.F., Dinwiddie D., Farrow E.G., Miller N.A., Favaretto F., Maffei P. (2015). Alström syndrome: Mutation spectrum of *ALMS1*. Hum. Mutat..

[33] Marshall J.D., Maffei P., Beck S., Barrett T.G., Paisey R.B. (2011). Clinical utility gene card for: Alström syndrome. Eur. J. Hum. Genet..

[34] Sukumolanan P., Petchdee S. (2022). Prevalence of cardiac myosin-binding protein C3 mutations in Maine Coon cats with hypertrophic cardiomyopathy. Vet. World..

[35] Korobova V., Kruglova Y. (2024). Influence of clinical aspects and genetic factors on feline HCM severity and development. Vet. Sci..

[36] Vandroux D., Aboyans V., Houehanou Y.C., Chastaingt L., Saka D., Sonou A., Amidou S., Houinato D., Preux P.M., Magne J. (2024). Impact of Hypertension on Left Ventricular Geometry and Diastolic Function in Africa: Results from the Population-Based TAnve Health (TAHES) Cohort Study. Am. J. Cardiol..

[37] Gaia de Sousa F., Mendes A.C.R., Carvalho L.P.d., Beier S.L. (2025). Clinical-diagnostic and therapeutic advances in feline hypertrophic cardiomyopathy. Vet. Sci..

[38] Casamian-Sorrosal D., Chong S., Fonfara S., Helps C. (2014). Prevalence and demographics of the *MYBPC3*-mutations in Ragdolls and Maine Coons in the British Isles. J. Small Anim. Pract..

[39] Godiksen M.T., Granstrøm S., Koch J., Christiansen M. (2011). Hypertrophic cardiomyopathy in young Maine Coon cats caused by the p. A31P cMyBP-C mutation-the clinical significance of having the mutation. Acta Vet. Scand..

