# Supplementary material for: Prevalence and Clinical Relevance of Alström Syndrome Protein 1 Gene Variant and Feline Hypertrophic Cardiomyopathy in Sphynx Cats in Thailand
Source: Animals (Basel). 2026 Jun 12;16(12):1815. doi: 10.3390/ani16121815 (PMC13296244; doi:10.3390/ani16121815)
Supplement: Supplementary file 1 [file animals-16-01815-s001.zip › animals-4314189-supplementary.pdf]

Supplementary Table

Supplementary Table S1. Baseline demographic and signalment data of the enrolled Sphynx cats.

Variable	Distribution
Sex, n (%)	
Female	25 (53.2)
Male	22 (46.8)
Age (month), median (IQR)	24 (12-30)
Age (categorical period), n (%)	
Kitten, 0-6 months	1 (2.1)
Junior, 7-24 months	28 (59.6)
Adult, 25-72 months	17 (36.2)
Mature, 73-120 months	1 (2.1)
Senior, 121-168 months	0
Geriatric, >168 months	0
Body weight (kg), mean (SD)	3.82 (0.94)
BCS, median (IQR)	5.75 (5-6)
BCS (categorical score), n (%)	
Emaciated, score 1	0
Underweight, score 2-3	2 (4.3)
Ideal, score 4-5	21 (45.7)
Overweight, score 6-7	23 (50.0)
Obese, score 8-9	0

Supplementary Table S2. Distribution of clinical characteriatics according to sex, age group, and body condition score in enrolled Sphynx cats.

Variable	Distribution, mean (SD)		
	**SBP (mmHg)**	**HR (bpm)**	**RR (bpm)**
Sex			
Female	150.66 (29.22)	202.00 (45.64)	52.32 (14.21)
Male	153.83 (25.13)	202.24 (48.13)	51.76 (15.87)
Age (period)			
Kitten, 0-6 months	N/A	115 (N/A)	80 (N/A)
Junior, 7-24 months	143.83 (25.37)[a]	201.52 (49.15)	51.63 (12.99)
Adult, 25-72 months	166.56 (25.08)[a]	210.60 (38.25)	50.93 (17.10)
Mature, 73-120 months	141.5 (N/A)	174.75 (N/A)	N/A
BCS (range score)			
Underweight, score 2-3	150 (26.16)	186 (50.91)	60 (5.66)
Ideal, score 4-5	151.72 (27.51)	192.41 (49.08)	48.05 (15.16)
Overweight, score6-7	152.53 (28.53)	213.08 (42.72)	55.50 (14.82)

Multivariable linear regression was used to examine factors associated with SBP, HR, and RR.

[a] mean difference (SD) = 24.18 (8.38), 95%CI = 7.24-41.12, p=0.006.

Supplementary Table S3. Distribution of echocardiographic characteristics and positive HCM phenotype according to sex, age group, and body condition score in enrolled Sphynx cats.

**Variable**	**Distribution**						
	**IVSd (mm), mean (SD)**	**LVPWd (mm), mean (SD)**	**LA/AO ratio, median (IQR)**	**MV E/A ratio, median (IQR)**	**IVRT (ms), median (IQR)**	**MV E/E' ratio, median (IQR)**	**Positive phenotype, n (%)**
Sex							
Female	0.44 (0.06)	0.43 (0.06)	1.44 (1.33-1.55)	1.08 (0.99-1.19)	44.00 (38.00-47.00)	9.45 (7.23-11.82)	1 (25.0)
Male	0.47 (0.09)	0.48 (0.12)	1.34 (1.32-1.46)	1.13 (1.06-1.30)	42.06 (38.00-49.00)	7.86 (6.11-9.71)	3 (75.0)
Age (period)							
Kitten	0.38 (N/A)	0.40 (N/A)	1.32 (N/A)	0.90 (N/A)	42.00 (N/A)	21.03 (N/A)	0
Junior	0.44 (0.06)	0.43 (0.06)	1.42 (1.34-1.54)	1.09 (1.02-1.22)	42.50 (37.90-46.90)	8.72 (7.12-10.48)	1 (25.0)
Adult	0.48 (0.08)	0.50 (0.13)	1.34 (1.44-1.32)	1.13 (1.04-1.20)	44.00 (38.00-51.00)	7.98 (7.01-9.46)	2 (50.0)
Mature	0.59 (N/A)	0.62 (N/A)	1.68 (N/A)	0.78 (N/A)	49.00 (N/A)	4.50 (N/A)	1 (25.0)
BCS (range score)							
Underweight	0.44 (0.07)	0.40 (0.04)	1.50 (1.41-1.60)	1.15 (1.09-1.21)	44.00 (44.00-44.00)	15.44 (13.43-17.45)	0
Ideal	0.45 (0.06)	0.44 (0.07)	1.34 (1.32-1.44)	1.05 (0.92-1.20)	45.00 (41.00-51.00)	8.78 (7.43)-10.15)	1 (25.0)
Overweight	0.47 (N/A)	0.48 (0.12)	1.43 (1.32-1.55)	1.13 (1.06-1.21)	41.00 (36.72-45.00)	8.38 (6.93-10.25)	3 (75.0)

Supplementary Table S4. The analysis of multivariable logistic regression for relation of characteristic variables to positive phenotype.

Clinical variable	Positive HCM phenotype		
	**Odds ratio**	**95% CI**	***p* Value**
Sex			
- Female	1	Reference	
- Male	1.62	0.11 – 22.77	0.720
Age (period)			
- Junior	1	Reference	
- Adult	3.78	0.29 – 49.37	0.310
- Mature	1	N/A	N/A
- Kitten	1	N/A	N/A
BCS (range score)			
- Ideal	1	Reference	
- Overweight	2.03	0.14 – 29.44	0.604
- Underweight	1	N/A	N/A
